# Remission of Abdominal Migraines Following Chiropractic Care for Thoracic Scoliosis

**DOI:** 10.7759/cureus.89887

**Published:** 2025-08-12

**Authors:** Eric Chun-Pu Chu

**Affiliations:** 1 Chiropractic and Physiotherapy Center, New York Medical Group, Hong Kong, CHN

**Keywords:** abdominal migraine, chiropractor, headadche, migraine, pediatric headache

## Abstract

Abdominal migraine (AM) is a pediatric functional gastrointestinal disorder characterized by recurrent episodes of abdominal pain and associated symptoms, for which existing pharmacological therapies may be limited by suboptimal efficacy and adverse effects, underscoring the need for alternative treatment approaches. This case report describes a 10-year-old girl diagnosed with mild thoracic scoliosis (20° Cobb angle) who complained of recurrent periumbilical pain for the past 16 months that lasts between two and 24 hours, accompanied by vomiting and pallor. These episodes were triggered by stress, lack of sleep, and skipped meals, and they provided minimal relief with ibuprofen. All her blood tests, ultrasound, and physical examinations returned negative results. Chiropractic examination revealed postural imbalances, thoracic tenderness, restricted motion, and normal neurological/physical findings. An impression of abdominal migraine prompted a treatment plan of spinal manipulation, axial traction, and soft tissue. By the second month, AM attacks stopped; a six-month X-ray indicated a reduction in scoliosis to 5° and an improvement in pelvic balance, which remained consistent at the 11-month mark. AM is a clinical diagnosis and is under-recognized. This novel case indicates that chiropractic care may serve as a promising non-pharmacological option, calling for further trials to assess its effectiveness against traditional therapies and to investigate processes such as gut-brain axis modulation in the management of abdominal migraines.

## Introduction

Abdominal migraine (AM) is a debilitating condition, affecting about 1% to 4% of the pediatric population but often overlooked in adults due to a lack of reports [1]. AM is characterized by paroxysmal, recurrent periumbilical pain associated with vasomotor symptoms such as pallor, nausea, vomiting, anorexia, and occasionally, photophobia and headache [1,2]. AM is a clinical diagnosis and is under-recognized. The recurrence of migraine in family histories suggests a genetic component to its predisposition [2]. Currently, the management of AM largely mirrors that of migraine headaches, primarily utilizing medications such as non-steroidal anti-inflammatory drugs (NSAIDs), antiemetics, and triptans for acute episodes, and prophylactic treatments including beta-blockers, calcium channel blockers, and antiepileptic drugs [2]. While these approaches can be effective, they are sometimes accompanied by side effects like medication overuse, headaches, and other adverse systemic reactions, which are particularly concerning in children [3]. Furthermore, a considerable portion of patients either fails to respond sufficiently to conventional treatments or suffers from relapses, highlighting the necessity for alternative therapeutic solutions [3].

Literatures suggest that non-conventional regimens such as chiropractic, physiotherapy, and massage therapy are effective in managing migraine headaches [4]. There is no case report describing chiropractic treatment for AM in the existing literature. This case report aims to enrich the discussion and prompt a more integrative approach to the management of AM, potentially fostering an alternative strategy for improving patient outcomes. This case report seeks to enhance the awareness and recognition of AM while encouraging a more holistic strategy for its treatment.

## Case presentation

Patient presentation

A 10-year-old underweight girl presented to the chiropractic clinic in June 2024 with a chief complaint of mild scoliosis and back stiffness, first noted by her parents during a routine school physical six months prior. Although the scoliosis was mild, it caused noticeable asymmetry in her posture and occasional discomfort during prolonged sitting or physical activities, such as sports. Additionally, she reported a history of recurrent moderate to severe stomach pains for the past 16 months. These episodes, lasting from two to 24 hours, were characterized by a dull ache localized around the umbilicus, rated between five and eight on the numeric pain scale. Associated symptoms included nausea, vomiting, loss of appetite, and noticeable pallor. Importantly, she felt completely normal between episodes, with no residual fatigue or gastrointestinal issues. The pain was significant enough to cause her to miss school for a total of eight days over the past six months, disrupting her education and social activities. Triggers included poor sleep, travel, motion sickness, skipped meals, and stress from academic pressures or family life.

Past medical history

The patient's medical history was otherwise unremarkable, with no chronic illnesses, allergies, or migraines. A review by a pediatrician included blood tests and an abdominal ultrasound, both of which ruled out common gastrointestinal pathologies such as appendicitis or inflammatory bowel disease. Over the past 16 months, she had been taking non-steroidal anti-inflammatory drugs (NSAIDs) like ibuprofen, which provided only temporary relief, suggesting limitations of pharmacological treatment for conditions potentially linked to neurogenic or spinal causes.

Physical examinations

**Figure 1:**
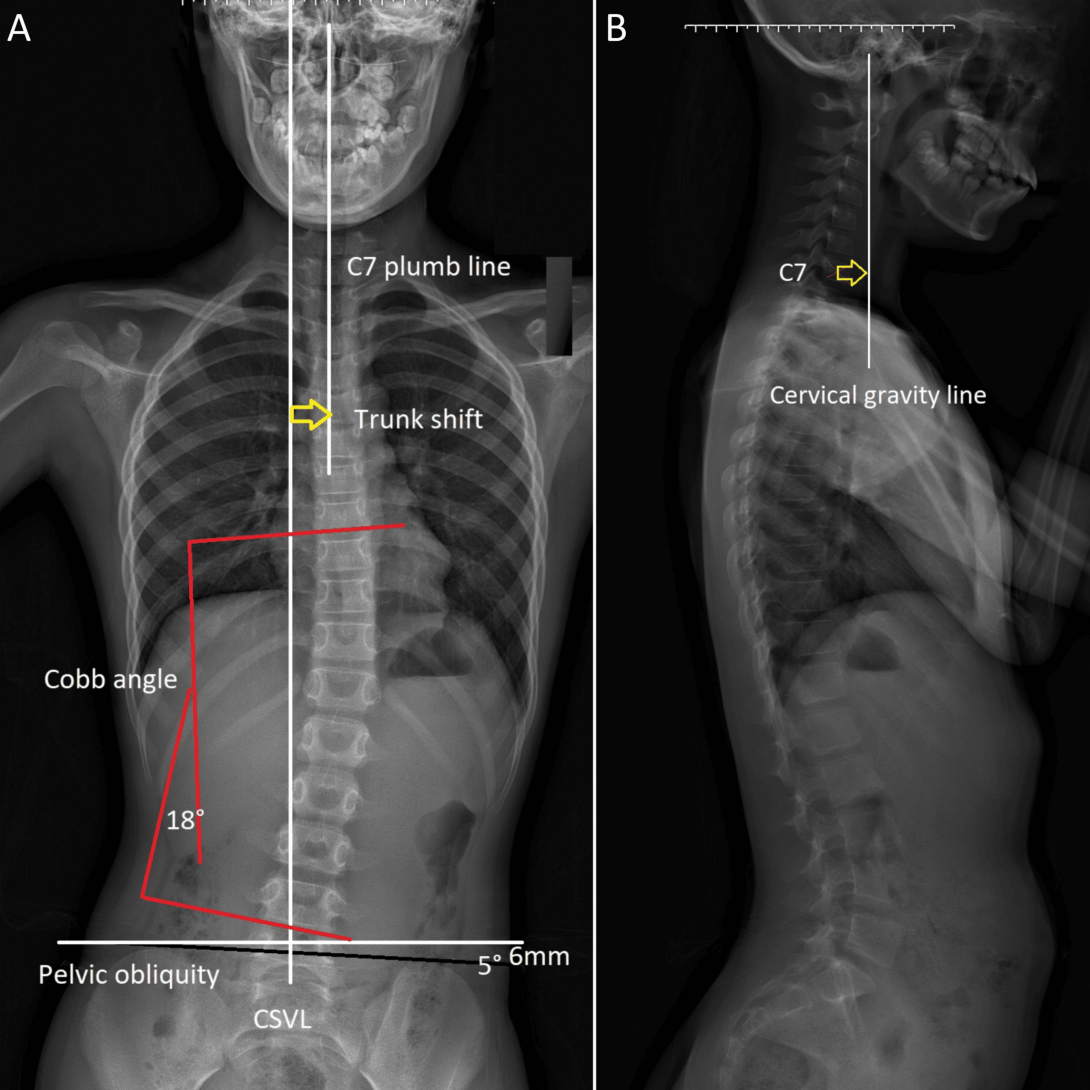
Full spine radiographs before scoliosis treatment. (A) Anteroposterior (AP) view: The Cobb angle was measured at 18°. The C7 plumb line is deviated from the central sacral vertical line (CSVL), indicating a trunk shift, and pelvic obliquity was 5°, with a pelvic imbalance of 6 mm. (B) Lateral view: The cervical gravity line is shifted anterior to the C7 vertebral body, consistent with forward head posture.

A comprehensive physical examination was conducted to evaluate the relationship between her scoliosis and back stiffness. Postural analysis revealed mild thoracic scoliosis with rightward convexity, forward head posture, and increased thoracic kyphosis, contributing to her reported stiffness. Palpation showed tenderness along the mid-thoracic paraspinals (T6-T10) and lumbar region (L2-L4), with hypertonicity in the erector spinae and quadratus lumborum muscles, but no acute injury signs. Range of motion testing indicated restricted thoracic extension (20° vs. normal 30-40°) and lumbar flexion (40° vs. normal 50-60°), limited by stiffness rather than pain. Neurological examination was unremarkable. An abdominal examination revealed mild tenderness in the periumbilical region, consistent with her pain description, but no rebound tenderness or guarding. A full-spine X-ray was ordered, revealing an 18° Cobb angle in the thoracic curve with vertebral rotation, but no congenital anomalies. The findings suggested that spinal misalignment could result in nerve interference and muscle imbalances, potentially causing visceral pain. As the patient fits into both the Rome IV and ICHD-3 diagnostic criteria, abdominal migraine was suspected (Figure 1).

Treatment

The treatment plan aimed to address both scoliosis and back stiffness. Initial management focused on correcting biomechanical imbalances through manual adjustments (high-velocity, low-amplitude thrusts) to the thoracic and lumbar spine, axial traction to improve spinal alignment, and soft tissue therapies (e.g., myofascial release) to alleviate muscle hypertonicity. Sessions were scheduled three times per week for the first month to expedite symptom relief and postural correction, utilizing gentle techniques suitable for a pediatric patient to avoid overstimulation of the autonomic system. Home exercises included core strengthening (e.g., planks and bird-dog poses) and stretching to support spinal stability, with parental guidance to minimize triggers such as poor sleep or skipped meals.

Outcome

**Figure 2:**
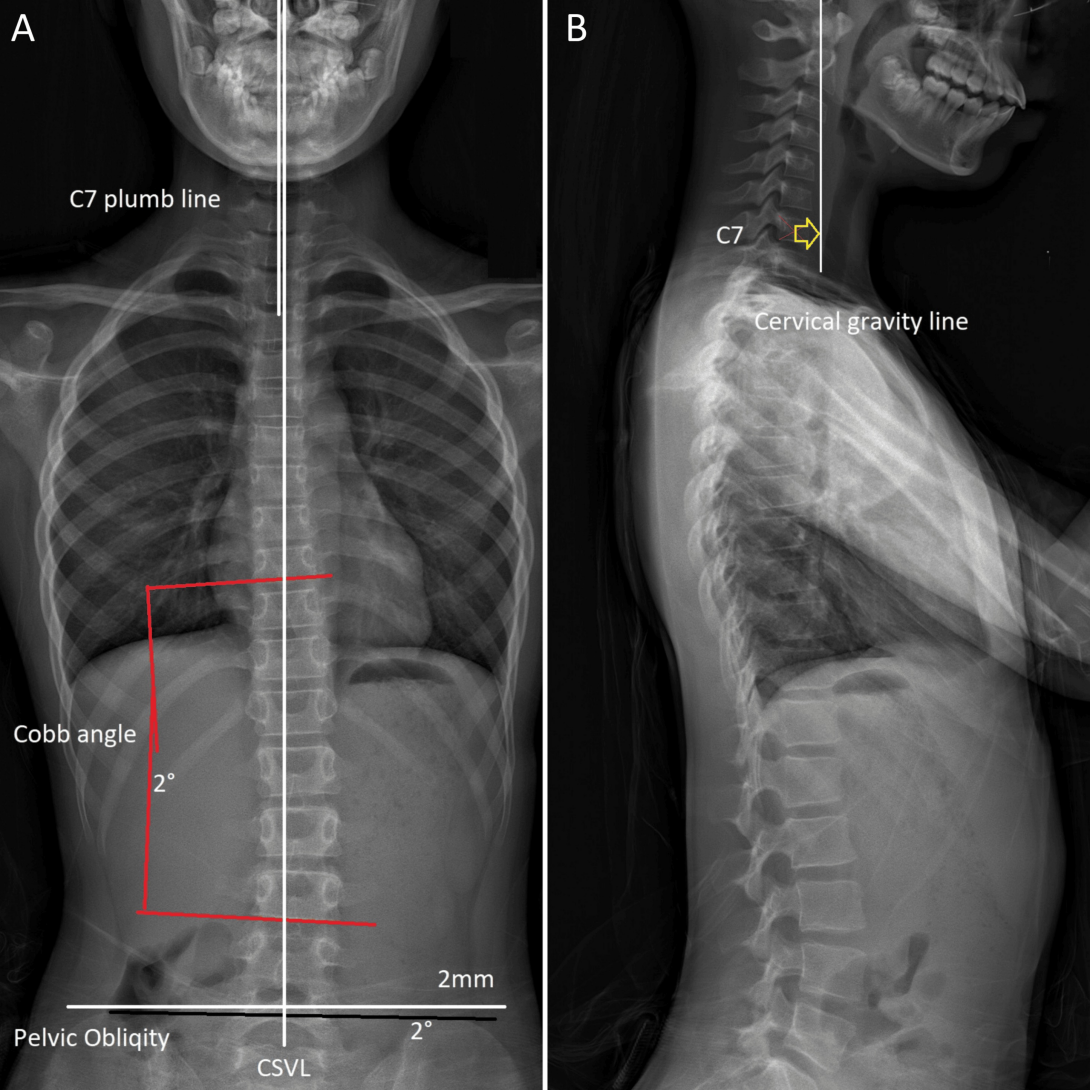
Full spine radiographs after scoliosis treatment. (A) Anteroposterior (AP) view: The Cobb angle improved from 18° to 2°. The C7 plumb line shifted closer to the central sacral vertical line (CSVL), indicating improved trunk alignment. Pelvic obliquity decreased from 5° to 2°, with pelvic imbalance reduced to 2 mm. (B) Lateral view: The cervical gravity line is less anterior to the C7 vertebral body than before treatment, reflecting improvement in forward head posture.

By the second month of treatment, the frequency of stomach pain episodes had reduced significantly, and they ceased altogether, correlating with restored spinal function and autonomic balance. As symptoms improved, treatment frequency was reduced to two times per week for two months, incorporating biofeedback for stress management to address potential autonomic triggers of abdominal migraine. Progress was monitored through pain diaries and functional assessments (e.g., school attendance). At the three-month mark, treatment was tapered to once per week for three months, with a follow-up X-ray showing significant improvements in scoliosis (Cobb angle reduced from 18° to 2°), balanced pelvic alignment, and resolution of back stiffness (Table 1). Throughout the treatment process, safety monitoring was conducted to assess any potential side effects of the chiropractic interventions, ensuring the patient's well-being was prioritized. Maintenance care continued at one session per month, and at the 11-month follow-up, the patient remained symptom-free, with no recurrence of abdominal pain or progression of scoliosis (Figure 2).

**Table 1:** Summary of pre-treatment vs. post-treatment data for abdominal migraine.

	Baseline data	Post-treatment data
Cobb angle (thoracic scoliosis)	18	2
Postural analysis	Mild thoracic scoliosis with rightward convexity, forward head posture, increased thoracic kyphosis	Improved alignment, reduced asymmetry
Thoracic extension range of motion	20° (normal 30-40°)	Improved (normal range achieved)
Lumbar flexion range of motion	40° (normal 50-60°)	Improved (normal range achieved)
Back stiffness	Present	Resolved
Episodes of abdominal pain	Moderate to severe, 5-8 on pain scale, occurring regularly over 16 months	Ceased by second month
School absenteeism	Eight days in last six months	No absenteeism reported
Parental reports of symptoms	Felt completely normal between episodes	Significant improvement in overall well-being
Treatment frequency	Three times per week	Tapered to once per month

## Discussion

Brain-gut axis refers to the exchange of messages between the brain and various components of the gastrointestinal system, including the gut microbiota, mucosa, and immune system. There is an interaction between the central nervous system and the richly innervated gut in AM. The enteric nervous system, a subset of the autonomic nervous system, regulates gastrointestinal activity with little input from the brain or spinal cord. Disruption in the gut-brain axis and communication between the enteric and central nerve system may contribute to the link between migrainous characteristics and abdominal symptoms [1,5]. Conversely, autonomic neurons and neuroendocrine factors can modify gut behavior in response [1,5]. Pediatric functional abdominal pain disorders, currently referred to as disorders of gut-brain interaction, comprise irritable bowel syndrome, functional dyspepsia, abdominal migraine (AM), and functional abdominal pain.

AM is an under-recognized cause of recurrent abdominal pain in children but has been largely overlooked in adults [1,5]. Epidemiologically, AM affects about 1% to 4% of the pediatric population. The pathophysiology of AM involves a combination of three factors: visceral hypersensitivity, alterations in the gut-brain enteric nervous system, and psychological factors [1,2]. Firstly, the visceral hypersensitivity leads to an abnormal secretion of excitatory neurotransmitters like serotonin, which not only influences gastrointestinal sensation, contributing to abdominal pain, but also affects motility and secretion within the gastrointestinal system. Secondly, research indicates that decreased gut motility may play a role in the manifestation of AM. Thirdly, psychological factors have been linked to more sensitive visceral perception [2]. This increased sensitivity may be exacerbated by stress-induced changes in the hypothalamic-pituitary-adrenal axis, enhancing central nervous system arousal and further dysregulating gastrointestinal function [2]. Thus, the interplay between physical and psychological factors underscores the complexity of abdominal migraine, necessitating a multifaceted approach to diagnosis and treatment.

Spinal manipulation has been used in correcting scoliosis at the chiropractic clinics [6-8]. In this case, spinal manipulation offers relief for AM through its potential impact on the gut-brain enteric nervous system and the modulation of visceral hypersensitivity. The premise is that spinal misalignments can disturb the normal functioning of the nervous system, including the autonomic nervous system that extensively innervates the gastrointestinal tract [9]. By correcting these misalignments, spinal manipulation can potentially relieve spinal nerve root compression, thereby modifying abnormal neuronal excitability and the excessive release of neurotransmitters such as serotonin that contribute to visceral hypersensitivity [10]. This adjustment may help normalize the altered gut motility and visceral pain pathways observed in AM. Furthermore, 15-months of chiropractic care might also mitigate stress-related exacerbations of symptoms by promoting relaxation and reducing sympathetic nervous system activity, which is often heightened in stress conditions. Through these mechanisms, chiropractic care not only addresses the direct neurological dysfunctions associated with AM but also tackles the broader psychosomatic elements, potentially offering a holistic improvement in symptoms.

This case of AM responding to spinal manipulation adds a novel dimension to the existing treatment strategy, which predominantly focuses on pharmacological and dietary management options. In comparison to other documented cases, this study underscores the potential of chiropractic interventions in managing neurogastroenterological conditions, a concept that has been less explored in conventional AM treatments. The effectiveness of spinal manipulation in this case suggests an intriguing link between spinal health and gastrointestinal function, possibly mediated by the autonomic nervous system. This connection is scarcely addressed in current AM research, marking a potential area for groundbreaking studies. Future research could include controlled clinical trials to rigorously evaluate the efficacy of spinal manipulation compared to standard pharmacological treatments for AM. Additionally, physiological studies could delve deeper into the mechanisms by which spinal manipulations influence gastrointestinal function and visceral sensitivity, potentially identifying biomarkers of response to chiropractic care. Such studies would not only validate or refute the findings of this case but also potentially expand the therapeutic repertoire for AM, offering relief to patients who might not respond to traditional treatments.

## Conclusions

This case suggests a potential benefit of the possibility of the therapeutic potential of spinal manipulation in treating AM, a condition that is often treated with medication and lifestyle changes. The resolution of symptoms following chiropractic manipulations suggests a possible interplay between spinal misalignment and gastrointestinal symptoms, possibly mediated by the autonomic nervous system. These findings introduce a novel perspective into the pathophysiology of AM, emphasizing the role of neuro-musculoskeletal health in managing gastrointestinal disorders. While the results are promising, we acknowledge the lack of standardized outcome tools in this study, which limits the objectivity of our findings. Future research should aim to incorporate validated instruments to enhance the assessment of treatment efficacy. If substantiated through further research, these insights could lead to broader treatment options that include spinal manipulation alongside conventional methods, offering a more holistic approach to patient care. Future research is required to confirm these findings and define the mechanisms by which spinal health influences gastrointestinal function, potentially guiding more effective and personalized treatment plans for AM sufferers.
